# An Efficient Leaching of Palladium from Spent Catalysts through Oxidation with Fe(III)

**DOI:** 10.3390/ma12081205

**Published:** 2019-04-12

**Authors:** Yunji Ding, Huandong Zheng, Jiayi Li, Shengen Zhang, Bo Liu, Christian Ekberg

**Affiliations:** 1 Institute for Advanced Materials and Technology, University of Science and Technology Beijing, Beijing 100083, China; dingyunji@163.com (Y.D.); zhenghuandongUSTB@163.com (H.Z.); ljy10278600@163.com (J.L.); 2 Nuclear Chemistry and Industrial Material Recycling, Department of Chemistry and Chemical Engineering, Chalmers University of Technology, 41296 Gothenburg, Sweden; che@chalmers.se

**Keywords:** spent catalysts, Palladium recovery, Fe^3+^ oxidative leaching, hydrometallurgy

## Abstract

Reclamation of spent catalysts for the efficient recovery of palladium (Pd) is gaining growing attention due to its scarcity and high supply risk. Currently Pd extraction from spent catalysts through an efficient, economical, and green method has remained a challenge. In this study, Fe^3+^ is utilized for leaching through oxidation of Pd in a mild condition. Before leaching, distillation was proposed to remove and recover the organics from spent catalysts. The effects of HCl concentration, Fe^3+^ concentration, NaCl concentration, leaching time, and temperature on the leaching efficiency of Pd were investigated to determine the optimum leaching conditions. The results show that Pd extraction and dissolution of Al_2_O_3_ increase with higher HCl concentration. The effect of NaCl on Pd leaching efficiency is significant at low acid concentration (2.0 mol/L HCl). The leaching efficiency was 99.5% for Pd under the following conditions: 2.0 mol/L HCl, 4.0 mol/L NaCl, and 0.67 mol/L Fe^3+^ at 80 °C for 90 min. The leaching kinetics fits best to the shrinking-core model of surface chemical reaction. The activation energy for the leaching of Pd was 47.6 kJ/mol. PdCl_4_^2−^ was selectively adsorbed by anion exchange resin. The filtrate containing adequate H^+^, Cl^-^, and Fe^3+^ was reused as leaching agent. Pd leaching efficiency was over 96% after five cycle times. This study provides an efficient process for recovery of Pd from spent catalysts.

## 1. Introduction

Palladium (Pd) plays a critical role in modern industry, especially in the field of catalysts [1,2,3]. According to the United States Geological Survey, the global consumption of Pd reached to 291 tons in 2016 and keeps increasing along with the development of technologies [4]. About 66% of Pd was used in catalytic industry, such as chemical and petroleum refining, automotive catalysts [5]. However, the reserve of Pd is limited and highly concentrated in South Africa, Zimbabwe, Russia, Canada, and United States, which endangers the supply to other countries and regions [6]. Therefore, it is significant to recover Pd from spent catalysts [7,8]. Particularly, recovery of one kilogram of Pd from spent catalysts can save about 45% of the energy costs and reduce 400 m^3^ water consumption compared with mining primary ores [9].

Although the content of Pd loaded on the catalysts is only approximately 0.05–1.0 wt. %, it is the dominant attraction of recycling spent catalysts due to the high economic value [8]. Pyrometallurgy and hydrometallurgy are typical technologies for precious metals recycling [10,11]. In the pyrometallurgical processes, spent catalysts are smelt with flux at high temperature by using Cu/Ni/Pb/Fe to collect precious metals [12,13,14]. Then, precious metals are recovered by chemical dissolution and refining from collector-alloy [15]. However, the defects including huge investment, high energy consumption, potential hazardous gas emissions and generation of a large quantity of slag, limit the flexibility of pyrometallurgical processes.

To avoid the disadvantages of a pyrometallurgical process, hydrometallurgical technologies have raised great attention [16]. However, the leaching efficiency of Pd is not satisfying in an ordinary leaching environment due to its high chemical stability [17]. Therefore, aggressive acid leaching with oxidizing agents (such as HNO_3_, NaClO_3_, and H_2_O_2_) are usually applied [18]. The leaching efficiency of Pd was over 99% with HCl in the presence of H_2_O_2_ at 90 °C [19], whereas 97% of Pd can be leached with a mixture of NaClO, H_2_O_2_, and HCl [20]. Trinh et al. recovered over 95% of Pd in 2.0 mol/L HCl by using 1.5 mol/L NaClO_3_ with HCOOH pre-reduction before leaching [21]. Kim et al. recovered 94% of Pd from spent automobile catalysts by introducing Cl_2_ in the concentrated HCl (6.0 mol/L) [22]. The use of Cu^2+^ as the oxidizing agent in HCl solution also yielded a high leaching efficiency of Pd (94.9%) [23]. However, these methods cause serious environmental issues due to the formation of hazardous gases (Cl_2_, NO_x_), acid fog, unconsumed acid, heavy metal ions (Pb^2+^, Cu^2+^, etc.), and large amounts of wastewater. The contaminants may cause severe pollution to the soil and water and even health problems [24]. Although the microwave-assisted solvent extraction achieved more than 90% of platinum group metals within 10–100 s, the processing capacity limits its commercial application [25].

Herein, in order to eliminate the potential environmental risks associated with Pd recovery, we developed an environmentally friendly process to recycle Pd from spent catalysts, using Fe^3+^ as oxidant in diluted HCl media to leach Pd, selectively adsorbing Pd by anion exchange resins and reusing the leaching agent. This process can improve the recovery efficiency of Pd and reduce the emission of pollution. This study may also provide a prospective way for recovery of Pd from other secondary resources.

## 2. Materials and Methods

### 2.1. Materials

The spent catalysts were recycled from the production of hydrogen peroxide by anthraquinone process. The concentration of Pd in spent catalysts was 1589.2 g/t, which was determined by ICP-MS (inductively coupled plasma optical emission spectrometer, RQ ICP-MS, PerkinElmer instruments, Waltham, MA, USA). The main composition of spent catalysts after distillation pretreatment is characterized by X-ray fluorescence (XRF-1800, Shimadzu, Kyoto, Japan), as shown in Table 1. All the elements are calculated in the form of oxides. The chemicals (HCl_aq_ (37%), FeCl_3_·6H_2_O, NaCl, NH_4_Cl, NH_3 (aq)_) were of analytical grade (≥99.9%). All the solutions were prepared in deionized water.

### 2.2. Experimental Procedures

**Figure 1:**
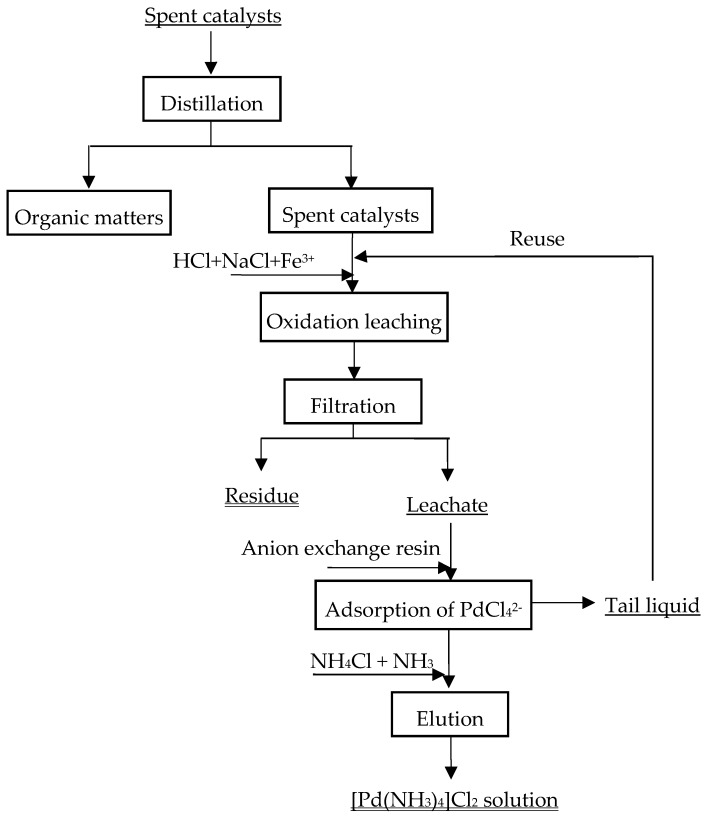
Flowsheet of leaching Pd from spent catalysts.

The flowsheet of Pd recovery is given in Figure 1. The recovery process is comprised of the following procedures.

(1) Distillation. Spent catalysts contain large amounts of organic matters, such as anthraquinones, aromatic solvent oil. Before leaching experiments, organic matters were separated by distillation at 250 °C, and then recovered through condensation.

(2) Fe^3+^ oxidation leaching. All the leaching experiments were carried out in a 400 mL beaker. The reactor was placed in a water bath to control the reaction temperature with magnetic stirring (300 rpm). The beaker was covered with plastic wrap to prevent the evaporation. 50 g of spent catalysts were put into the beaker, as well as the mixture solutions of FeCl_3_, HCl and NaCl at different concentrations. The solid to liquid ratio (S/L) was fixed to 1:5 g/ml. The following operating variables were studied: concentration of HCl (2.0–6.0 mol/L), Fe^3+^ (0–1.0 mol/L) and NaCl (0–4.0 mol/L), temperature of 40–80 °C for 30–120 min. After leaching, the residues and leachate were separated via filtering. The leachate was withdrawn to recover Pd through adsorption with anion exchange resins. Besides, all the experiments are independent.

(3) Reuse of tail liquid. After the leachate had passed through the column of anion exchange resins, Pd was recovered through adsorption. The tail liquid containing large amounts of Fe^3+^, H^+^, Cl^−^, and Fe^2+^ was reused as leaching agent. Before leaching spent catalysts, 1.0 ml H_2_O_2_ was added to oxide Fe^2+^ into Fe^3+^. HCl_(aq)_ was used to adjust the concentration of H^+^.

### 2.3. Chemical Analysis and Characterization

The concentration of Pd in the leachate was analyzed by inductively coupled plasma optical emission spectrometer (ICP-MS, Optima 8000, PerkinElmer instruments). All the aqueous samples were co-precipitated with 1.0 mol/L SnCl_2_ and 2.5 g/L TeCl_4_ for 30 min. The solid samples were completely digested by aqua regia at 120 °C for 240 min in the autoclave before co-precipitation. Then the precipitates were dissolved by aqua regia at 80 °C, and [Pd(NH_3_)_4_]Cl_2_ the obtained solution was concentrated to 0.1–0.5 mL. The concentrated solution was diluted by 0.1 mol/L HCl and transferred to a 50 ml volumetric flask. Finally, Pd was analyzed by ICP-MS. Leaching efficiency of Pd can be calculated by using Equation (1). The concentration of Al^3+^ was also analyzed by ICP-MS.

(1) $$\mathrm{Leaching}\;\mathrm{efficiency}\;\mathrm{of}\;\mathrm{Pd}=\frac{c_{1}\times v_{1}}{c_{0}\times m_{0}}\times 100\%,$$

where m_0_ (g/t) is the mass of spent catalysts after distillation and c_0_ (g/t) is the concentration of Pd in spent catalysts; c_1_ (mg/L) is the concentration of Pd in the leachate; v_1_ (mL) is the volume of leachate.

X-ray diffraction (XRD, Rigaku D/max-2550 V) was performed on the spent catalysts before and after distillation pretreatment, using Cu K_α1_ radiation at an operating voltage and current of 40 kV and 40 mA, respectively. The scan range was 2θ = 10–90°, and the scan speed was 20 °/min. Thermogravimetric analysis and differential scanning calorimetry (TG/DSC, SDT Q600, TA Instruments, New Castle, DE, USA) were utilized to determine the optimum temperature for distillation of the spent petrochemical catalysts. The recovered products by distillation were analyzed by FTIR spectroscopy (NEXUS670FT-IR, Thermo Nicoiet, Madison, USA). The solid product was mixed with KBr and pelletized for composition analysis, while the liquid product was dropped on the KBr pellets. The functional groups of the samples were detected between 4500 and 500 cm^−1^. The scans were performed with a resolution of 2 cm^−1^. The baseline of the raw data was adjusted and then the modified data was normalized.

## 3. Results and Discussion

### 3.1. Thermodynamics of Reactions

The dissolution of Pd is based on a high oxidation potential and effective complexing ions. Chloride ion is an excellent media for dissolution of Pd because of the formation of stable complex (PdCl_4_^2−^) and it decreases the standard reduction potential for electrode reactions [26]. The standard electrode potentials for the half reactions (with and without chloride ion) are given as follows [27,28]:

Pd^2+^ + 2e^−^ → Pd     ε^0^ = 0.951 V, (2)

[PdCl_4_]^2−^ + 2e^−^ → Pd + 4Cl^−^   ε^0^ = 0.621 V, (3)

where ε^0^ is the standard electrode potential. Compared with the standard potential of Pd in absence of chloride ion (ε^0^ = 0.951 V), the chloride ion decreases the potential drastically (ε^0^ = 0.621 V), which promotes the dissolution of Pd. According to the Nernst equation, the equilibrium potential (ε) of Pd depends on the ε^0^ and the activities (the concentrations as estimate) of ions in the solution.

(4) $$\varepsilon =\varepsilon^{0}+\frac{0.0592}{2}\mathrm{lg}\frac{{c(\mathrm{PdCl}}_{4}^{-})}{c^{4}{(\mathrm{Cl}}^{-})}$$

**Figure 2:**
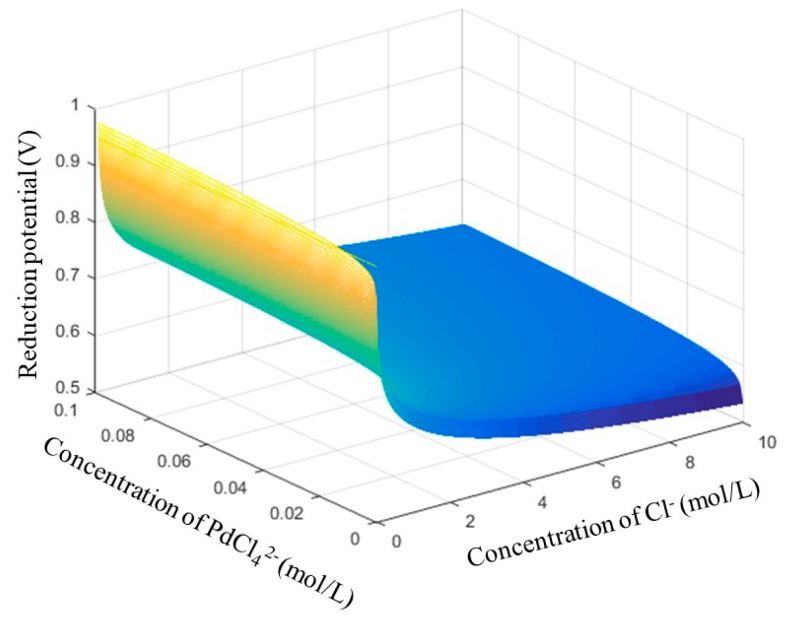
Equilibrium potentials for semi-reaction of Pd reduction in chloride media.

Obviously, the equilibrium potential decreases because of the increasing concentration of Cl^−^ and decreasing concentration of PdCl_4_^2−^, as shown in Figure 2. The equilibrium potential is only 0.532 V when the concentrations of Cl^−^ and PdCl_4_^2−^ are 10.0 mol/L and 0.01 mol/L, respectively. Figure S1 illustrates the standard potentials for several traditional oxidizing agents (e.g., NaClO_3_, HClO, HNO_3_, H_2_O_2_, Cl_2_, and Fe^3+^), indicating that they are potential oxidant candidates for Pd dissolution. Fe^3+^ is studied in this paper due to its non-toxicity, being friendly to the environment and the economy. The standard electrode potential for Fe^3+^/Fe^2+^ semi-reaction is as follows:

Fe^3^^+^ + e^−^ → Fe^2^^+^   ε^0^ = 0.77 V. (5)

The potential of Fe^3+^/Fe^2+^ is 0.77 V, which is above the potential curve of Pd. Therefore, Pd leaching reaction using Fe^3+^ as oxidizing agent is shown in reaction (6).

2Fe^3+^ + Pd + 4Cl^−^ → PdCl_4_^2−^ + 2Fe^2+^ (6)

Therefore, the potential (E) of the reaction is determined by the reactions of (5) and (6), as shown in Equation (7).

(7) $$E=E^{0}-\frac{0.0592}{2}\mathrm{lg}\frac{c({\mathrm{PdCl}}_{4}^{2-})\cdot c^{2}({\mathrm{Fe}}^{2+})}{c^{4}({\mathrm{Cl}}^{-})\cdot c^{2}({\mathrm{Fe}}^{3+})}\;(E^{0}=0.15\;V),$$

where *E^0^* is standard potential of the reaction. To confirm the oxidation of Pd(0), the relationship between concentrations of Cl^−^ and Fe^3+^ is shown in Equation (8) when the reaction reaches an equilibrium.

(8) $$0=0.15+0.0592\mathrm{lg}\frac{c({\mathrm{Fe}}^{3+})}{c({\mathrm{Fe}}^{2+})}-\frac{0.0592}{2}\mathrm{lg}\frac{c({\mathrm{PdCl}}_{4}^{2-})}{c^{4}({\mathrm{Cl}}^{-})}$$

**Figure 3:**
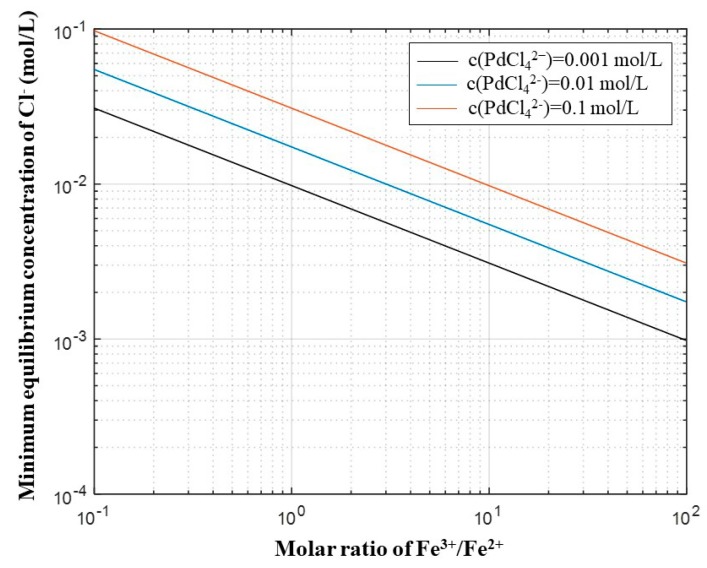
The relationship between the molar ratio of Fe^3+^/Fe^2+^ and the necessary concentration of chloride for oxidation of Pd to PdCl_4_^2−^.

According to the Equation (8), Figure 3 presents the relationship between the molar ratio of Fe^3+^/Fe^2+^ and the necessary concentration of Cl^−^ with constant PdCl_4_^2−^ concentrations of 0.1, 0.01, and 0.001 mol/L, respectively. The minimum equilibrium concentration increases with rising PdCl_4_^2−^ concentration, and decreases with higher molar ratio of Fe^3+^/Fe^2+^. The necessary concentration of Cl^−^ is shown in Figure 3. It is clear that the Pd leaching by Fe^3+^ is easy in chloride media theoretically. For example, the equilibrium concentration of Cl^−^ is lower than 0.1 mol/L when the concentration of PdCl_4_^2−^ varies from 0.001 to 0.1 mol/L, as well as the ratio of Fe^3+^/Fe^2+^ varying from 0.1 to 100.

### 3.2. Characterization of Spent Catalysts

**Figure 4:**
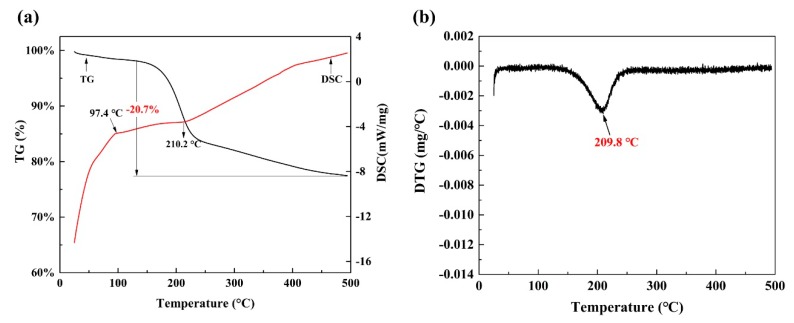
(**a**) Thermogravimetric analysis and differential scanning calorimetry (TG/DSC) curves and (**b**) DTG curve of spent catalysts.

The TG-DSC diagram shows the mass losses and two endothermic peaks, as seen in Figure 4a. The weight loss (1.9%) between 25–97 °C corresponds to the loss of free water. The peak at 210.2 °C corresponds to the volatilization of organic materials, which indicates a weight loss of 18.8% between 97–500 °C. The DTG (differential thermogravimetry) curve presented in Figure 4b shows the volatilization temperature of organics is 209.8 °C. Hence, the distillation temperature was set at 250 °C with the aim to increase the volatility of organics.

After 2 h distillation at 250 °C, the mass of the samples did not decrease along with extension of time. The main component of spent catalysts after distillation pretreatment is Al_2_O_3_, which accounts for over 92%. The contents of other elements, such as P_2_O_5_, CaO, and K_2_O, are only 4.38, 0.98, and 0.88%, respectively. The volatile organics were recovered after condensation (As seen in Figure S2). Figure S3 shows the XRD patterns of the spent catalysts, as well as the samples after distillation pretreatment. The results show that the main phase of both samples is θ-Al_2_O_3_, indicating crystal form was not changed by distillation. Other phases cannot be detected due to their low contents. The recovered products by distillation were characterized by FTIR spectroscopy in Figure S4, which were probably to be anthraquinones and aromatic solvent oil, respectively.

### 3.3. Fe^3+^ Oxidation Leaching

#### 3.3.1. Effect of HCl Concentration

**Figure 5:**
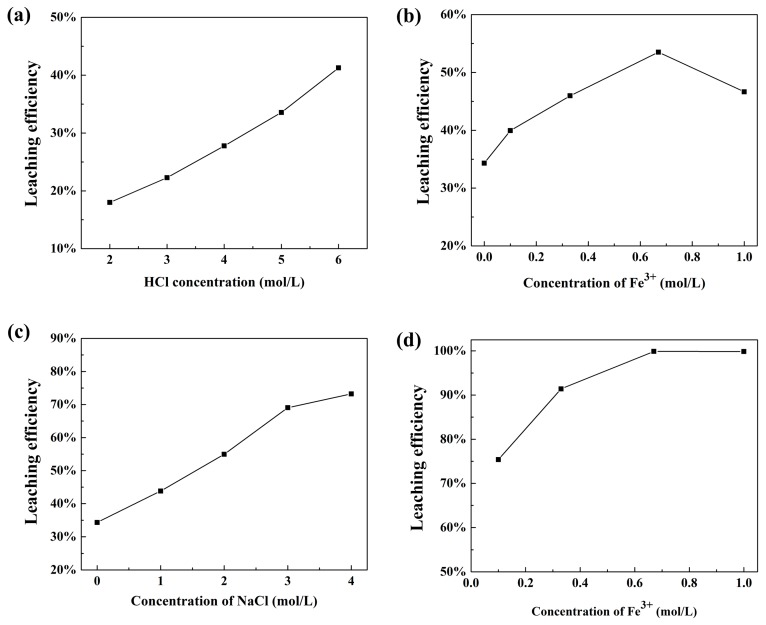
Effects on Pd leaching efficiency of (**a**) concentration of HCl, (**b**) concentration of Fe^3+^, (**c**) concentration of NaCl, and (**d**) concentration of Fe^3+^ with 4.0 mol/L NaCl.

The effect of HCl concentration on the leaching efficiencies of Pd and the dissolution of catalyst supports were investigated under the conditions: S/L of 1:5, at 60 °C for 120 min, as shown in Figure 5a. The concentration of HCl varied from 2.0 to 6.0 mol/L. Figure 5a showed that higher concentration of HCl improved the leaching efficiency of Pd and enhanced the dissolution of Al_2_O_3_. The leaching efficiency of Pd increased from 18.0 to 41.2% as HCl concentration increased from 2.0 to 6.0 mol/L. The weight loss of spent catalysts at different concentrations of HCl were shown in Table 2. Less Al_2_O_3_ was dissolved in lower concentration of HCl. Only 1.6% of the supports were dissolved when the concentration of HCl was 2.0 mol/L. The maximum weight loss for supports was 14.9% under the acid concentration of 6.0 mol/L. High dissolution of Al_2_O_3_ makes the separation of leaching residue and solution difficult and decreases the leaching efficiency of Pd. Therefore, 2.0 mol/L was determined as the concentration of HCl for the following experiments. The leaching efficiency of Pd can be enhanced by adding Fe^3+^, NaCl, and increasing leaching temperature.

#### 3.3.2. Effect of Fe^3+^ Concentration at 2.0 mol/L HCl

The effect of Fe^3+^ concentration was carried out by changing the initial concentrations of Fe^3+^ from 0 to 1.0 mol/L at the constant concentration of HCl 2.0 mol/L, S/L of 1:5, at 80 °C for 120 min. It could be found from Figure 5b that the concentration of Fe^3+^ exhibited significant effect on the extraction of Pd. With Fe^3+^ concentration increased from 0 to 0.67 mol/L, Pd leaching efficiency increased from 34.32 to 53.5%. The increased leaching efficiency of Pd occurred because Fe^3+^ ions acted as an oxidant to decrease the electrode potential of Pd complex. A further increase in the Fe^3+^ concentration to 1.0 mol/L did not cause significant change for Pd leaching. Therefore, 0.67 mol/L of Fe^3+^ was considered as the appropriate concentration.

#### 3.3.3. Effect of NaCl Concentration

As the leaching efficiency of Pd was not optimistic under the above leaching conditions. According to the above theoretical analysis, a higher concentration of Cl^−^ can promote the dissolution of Pd. Figure 5c shows the effect of NaCl concentration on the leaching efficiency of Pd under the conditions: HCl concentration of 2.0 mol/L, S/L of 1:5, temperature of 80 °C for 120 min. The leaching efficiency of Pd were found to increase with increasing NaCl concentration, which was in agreement with theoretical analysis. This can be attributed to the decrease of electrode potentials of Pd, which promotes its dissolution in acid solution. When the concentration of NaCl reached 4.0 mol/L, Pd leaching efficiency increased to 73.2%. However, the increasing tendency slowed with further increasing NaCl concentration. Therefore, 4.0 mol/L was determined as the optimal concentration of NaCl.

#### 3.3.4. Effect of Fe^3+^ Concentration at 4.0 mol/L NaCl

The effect of Fe^3+^ concentration with 4.0 mol/L of NaCl on the leaching efficiency of Pd was studied. The leaching conditions were: S/L of 1:5, HCl concentration of 2.0 mol/L, at 80 °C for 120 min. Figure 5d shows that Pd leaching efficiency increases with higher Fe^3+^ concentration. When the concentration of Fe^3+^ was 0.1 mol/L, Pd leaching efficiency was 75.4%. When the Fe^3+^ concentration increased to 0.67 mol/L, more than 99% of Pd was recovered. Compared with Figure 5b, a huge improvement of Pd extraction (46.4%) was achieved by adding 4.0 mol/L NaCl. Pd leaching efficiency increased from 73.2% to 99.9% with the addition of 0.67 mol/L Fe^3+^. The results show that both Fe^3+^ and NaCl promote the oxidation of Pd. Hence, 2.0 mol/L HCl, 4.0 mol/L NaCl and 0.67 mol/L Fe^3+^ was chosen as optimal leaching agent in the following experiments.

### 3.4. Kinetic Analysis of Pd Leaching

**Figure 6:**
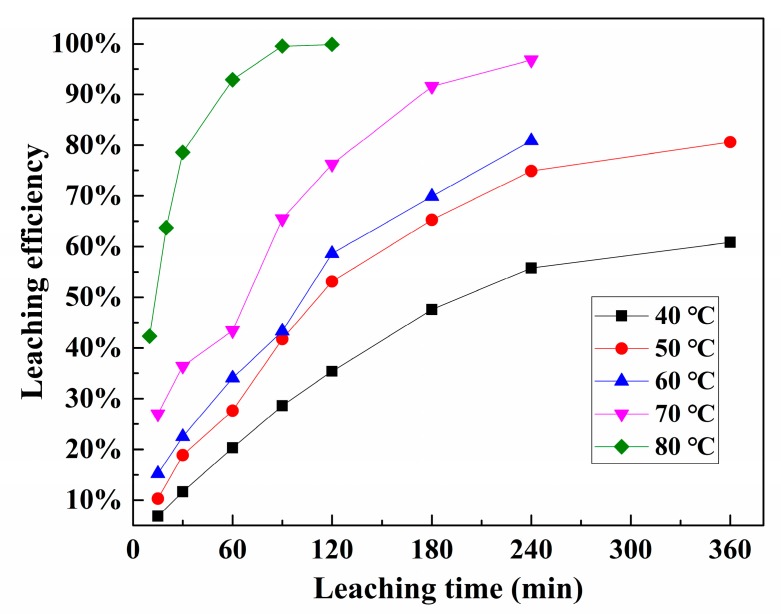
Leaching efficiency of Pd from spent catalysts under different temperatures and times.

The behavior of Pd leaching was studied for different reaction times (10–360 min) and temperatures (40–80 °C) under the following conditions: S/L of 1:5, Fe^3+^ concentration of 0.67 mol/L, HCl concentration of 2.0 mol/L, and NaCl concentration of 4.0 mol/L. The results are shown in Figure 6. The leaching temperature affected the Pd leaching efficiency significantly. Pd leaching efficiency increased as temperature rose, which was more obvious when the temperature was above 60 °C. This is because Pd leaching is an endothermic process. Furthermore, the average kinetic energy of the molecules increases with a rising temperature, which causes more frequent and more energetic collisions. Therefore, higher temperature can accelerate Pd leaching reaction. Pd leaching efficiency increased from 28.6% to 99.9% as temperature rose from 40 to 80 °C for 90 min. The leaching time also shows great impact on Pd leaching efficiency. For example, Pd leaching efficiency experienced a sharp increase with prolonged leaching time from 30 to 60 min at the temperature of 80 °C. Afterward, the leaching efficiency of Pd increased slowly and reached 99.5% with 90 min. The weight loss of spent catalysts was only 2.7% at 80 °C for 90 min.

Pd leaching is a solid-liquid heterogeneous reaction and considered to occur on the surfaces of unreacted particles. The shrinking-core model has been successfully applied to describe leaching kinetics. Pd leaching efficiency depends on the chemical reaction and the mass transfer (including inner and outer layer diffusion). Thus, Pd leaching efficiency can be assumed to be controlled by boundary layer mass transfer, surface chemical reaction or inert ash layer mass transfer. The leaching kinetics of the process can be described by the following expressions, respectively [29].

x = k_1_·t, (9)

1 − (1 − x)^1/3^ = k_2_·t, (10)

**Figure 7:**
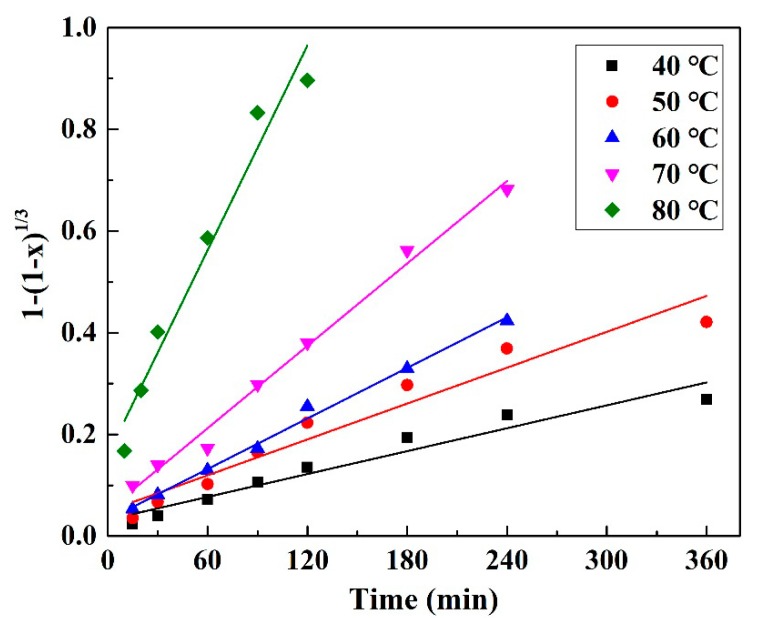
Plots of the shrinking-core model for chemical reaction control for Pd leaching.

1 − 3(1 − x) + 2(1 − x) = k_3_·t, (11)

where x is Pd leaching efficiency, k_1_, k_2_, and k_3_ are the reaction rate constant (min^−1^), and t is the reaction time (min). For example, if the largest resistance to Pd leaching is surface chemical reaction, the leaching kinetics of the process can be described by expression (10). The plot of 1 − (1 − x)^1/3^ versus t exhibits the best fitting relevance among the three models, as shown in Figure 7 (the results of Equations (9) and (11) are given in Figures S5 and S6 and Tables S1 and S2, respectively), indicating Pd leaching process using Fe^3+^ as oxidant is controlled by surface chemical reaction. The kinetic parameters at different temperatures are shown in Table 3.

**Figure 8:**
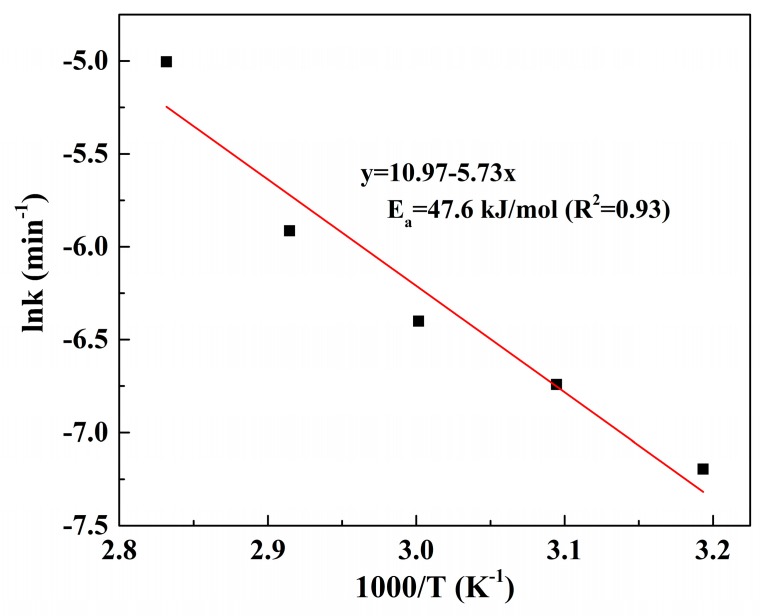
Arrhenius plot for the leaching of Pd in the temperature range 313.15–353.15 K.

The relationship between the reaction rate constant and temperature can be described by the Arrhenius equation.

ln k = ln A − E_a_/RT, (12)

where A is the pre-exponential factor, E_a_ is the apparent activation energy (kJ/mol), R is the universal gas constant (8.314 J/K/mol), and T is the absolute temperature (K). Plot of lnk versus 1/T is shown in Figure 8. The apparent activation energy for Pd leaching was 47.6 kJ/mol, indicating the process was controlled by surface chemical reaction.

### 3.5. Reuse of Leaching Agent

**Figure 9:**
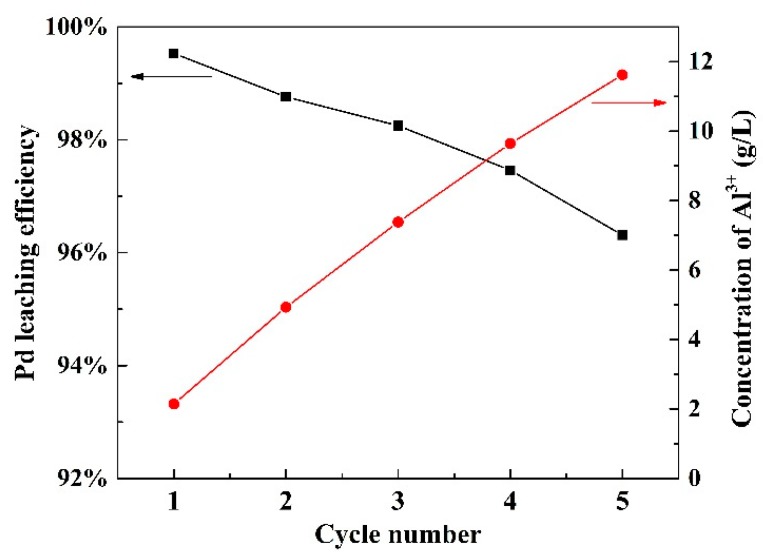
The effect of cycle number of leaching agent on Pd leaching and Al_2_O_3_ dissolution.

Anion exchange resins was used to selectively adsorb PdCl_4_^2−^ from leachate. The adsorption efficiency was 97.6% when leaching agent was composed of 0.67 mol/L Fe^3+^, 2.0 mol/L HCl, and 4.0 mol/L NaCl. The detailed information of adsorption of Pd and its elution can be found in the Supplementary Materials (Table S3–S6). After the leachate passed through the anion exchange resins, the tail liquid containing Fe^3+^, Fe^2+^, H^+^, and Cl^−^ was obtained. Before each cycle experiment, the concentration of H^+^ was adjusted to 2.0 mol/L and Fe^2+^ was oxidized into Fe^3+^ by 1.0 mL H_2_O_2_. Figure 9 shows that Pd leaching efficiency decreases slowly with cycle number. Pd leaching efficiency was 96.3% after five cycle times. The information of Pd leaching efficiency was shown in Table S7. Meanwhile, the accumulation of Al^3+^ in the solution increased from 2.14 g/L to 11.62 g/L. The increasing concentration of Al^3+^ increased the viscosity of leaching solution, which reduced the diffusion rate of reactants. This may be one of the reasons that resulted in the decrease of Pd leaching efficiency.

## 4. Conclusions

An efficient process was developed to recover Pd from spent catalysts. The organics including anthraquinones aromatic solvent oil have been recovered through low temperature distillation (at 250 °C). The mixture solution of NaCl and hydrochloric acid were employed as the leaching agent and FeCl_3_ as the oxidant. The optimum leaching conditions were determined to be HCl concentration of 2.0 mol/L, NaCl concentration of 4.0 mol/L, Fe^3+^ concentration of 0.67 mol/L, temperature of 80 °C, and leaching time of 90 min. Under the experimental conditions, the leaching efficiency of Pd was 99.5% and the dissolution of catalyst supports was only 2.7%. The increasing concentrations of NaCl, HCl, and Fe^3+^ can lead to a considerable enhancement of the leaching efficiency of Pd, so can leaching temperature and time. Kinetics analysis indicates that the Pd leaching process is controlled by surface chemical reactions. Compared with traditional aggressive acid leaching (e.g. aqua regia, HCl + NaClO_3_/H_2_O_2_), this process has eliminated the secondary pollution (toxic gases and heavy metal ions) by using Fe^3+^ oxidation leaching without sacrificing high leaching efficiency. Furthermore, the leaching agents can be reused after adsorption of PdCl_4_^2−^ by ion exchange resins. The leaching efficiency of Pd was 96.3% after five cycle times. This study provides a simple and efficient technology for recovery of Pd from spent catalysts.

## 5. Patents

The following patent was resulted from the work reported in this manuscript.

Shengen Zhang, Yunji Ding, Bo Liu. A green method for palladium recovery from scraps. Invention Patent, China. Appl. No. 201810670273.5, 2018.6.26.

## Figures and Tables

**Table 1:** The main elements content of spent catalysts analyzed by X-ray fluorescence (XRF).

Elements	Al_2_O_3_	P_2_O_5_	CaO	K_2_O	SiO_2_	Fe_2_O_3_	Na_2_O	TiO_2_	Others
Content (%)	92.85	4.38	0.98	0.88	0.42	0.13	0.097	0.059	0.204

**Table 2:** The weight loss of spent catalysts at different concentrations of HCl.

HCl_(aq)_/mol/L	2.0	3.0	4.0	5.0	6.0
Mass (g)	49.20	48.63	47.48	44.79	42.56
Weight loss (g)	0.8	1.37	2.53	5.21	7.44
Weight loss (%)	1.6%	2.7%	5.1%	10.4%	14.9%

**Table 3:** The kinetics parameters of chemical reaction control model for Pd leaching.

T	40 °C	50 °C	60 °C	70 °C	80° C
k	0.00075	0.0012	0.0017	0.0027	0.0067
R^2^	0.9322	0.9334	0.9924	0.9890	0.9565

## Supplementary Materials

The following are available online at https://www.mdpi.com/1996-1944/12/8/1205/s1, Figure S1: The reduction potentials for PdCl_4_^2−^ in chloride media at the concentration of 0.001, 0.01, and 0.1 mol/L. Figure S2: The organics recovered from spent catalysts by distillation. Figure S3: XRD patterns of the spent catalysts before and after distillation. Figure S4: The infrared spectra of recovered products by distillation, (a) solid product and (b) liquid product. Figure S5: Plots of x vs. t at different reaction temperatures. Figure S6: Plots of 1 − 3(1 − x)^2/3^ + 2(1 − x) vs. t at different reaction temperatures. Figure S7. Arrhenius plot for the leaching of Pd from spent catalysts calculated by the ash layer diffusion model. Table S1: Kinetics parameters during the leaching process calculated by the mass transfer control model. Table S2. Kinetics parameters during the leaching process calculated by the ash layer diffusion model. Table S3. The effect of Fe^3+^ on the adsorption efficiency of Pd by R_410_ ion exchange resins. Table S4. The effect of H^+^ on the adsorption efficiency of Pd by R_410_ ion exchange resins. Table S5. Elution efficiency of Pd from loaded resins by using different reagents. Table S6. The desorption efficiency of Pd by NH_4_Cl + NH_3_. Table S7. The leaching efficiency of Pd by reusing leaching agent.
