# Supplementary material for: An Efficient Leaching of Palladium from Spent Catalysts through Oxidation with Fe(III)
Source: Materials (Basel). 2019 Apr 12;12(8):1205. doi: 10.3390/ma12081205 (PMC6515284; doi:10.3390/ma12081205)
Supplement: Supplementary file 1 [file materials-12-01205-s001.pdf]

materials

MDPI

Supplementary Materials

An Efficient Leaching of Palladium from Spent Catalysts Through Oxidation with Fe(III)

Yunji Ding [1], Huandong Zheng [1], Jiayi Li [1], Shengen Zhang [1,*], Bo Liu [1,*] and Christian Ekberg [2]

[1] Institute for Advanced Materials and Technology, University of Science and Technology Beijing, Beijing 100083, PR China

[2] Nuclear Chemistry and Industrial Material Recycling, Department of Chemistry and Chemical Engineering, Chalmers University of Technology, 41296 Gothenburg, Sweden

* Correspondence: zhangshengen@mater.ustb.edu.cn (S.Z.); liubo@ustb.edu.cn (B.L.); Tel.: +86-010-62333375 (S. Z.)

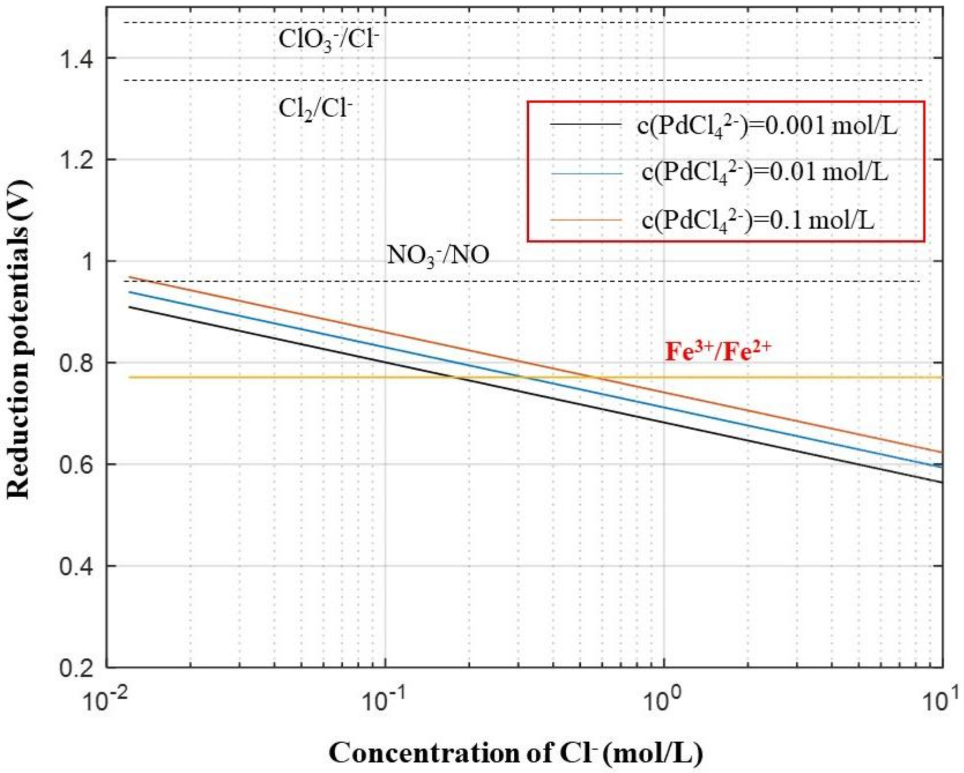

Figure S1. The reduction potentials for $PdCl_4^{2-}$ in chloride media at the concentration of 0.001, 0.01 and 0.1 mol/L.

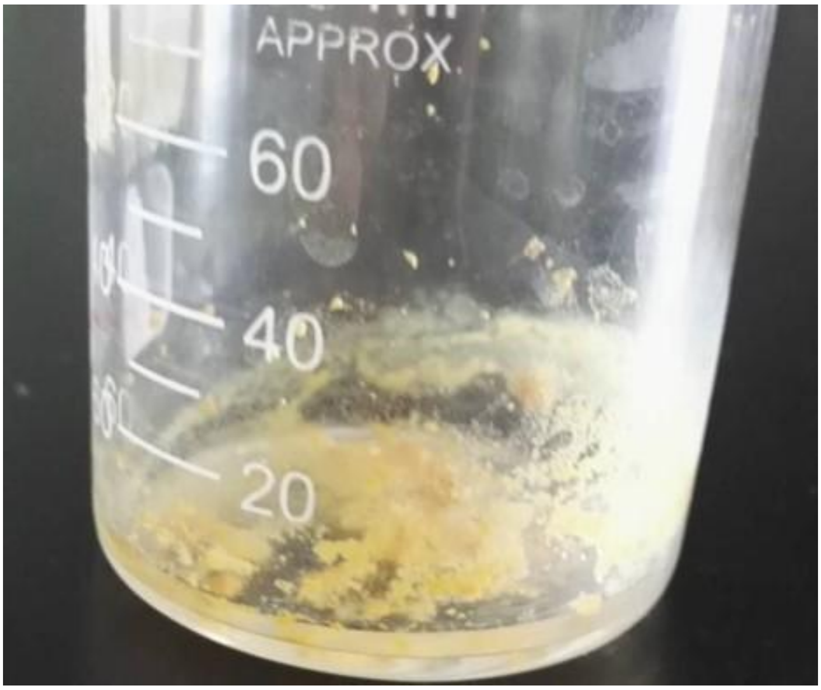

Figure S2. The organics recovered from spent catalysts by distillation.

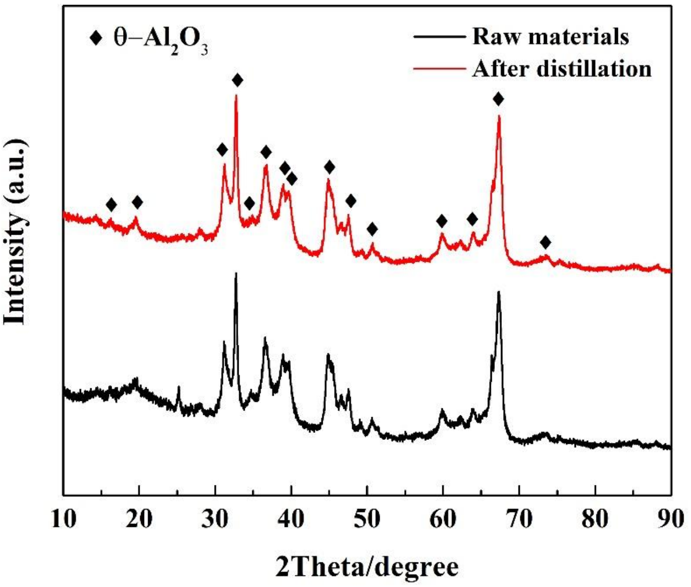

Figure S3. XRD patterns of the spent catalysts before and after distillation.

The FTIR spectroscopy was used to identify the functional groups of recovered products by distillation. The results are shown in Figure S4. The yellow solid distillage is probably to be anthraquinones, which contain many kinds of functional groups, such as –OH, $–CH_2OH$, $–CH_3$, –COOH, -C=O. The characteristic peak in Figure S4(a) at 3450.2 cm^{-1} is caused by the absorbance of –OH stretch. The peak at 2943.2 cm^{-1} is assigned to $-CH_3$ and $-CH_2OH$ vibrations. The bands at 1666.4 and 1593.1 cm^{-1} are assigned to –C=C and –C=O vibrations. The bands in the range of 1550 -700 cm^{-1} include the –C-C stretching and –C-O bending vibrations. The characteristic peaks are consistent with the functional groups of anthraquinones. The characteristic peaks (3477.4, 1645.2 and 715.5 cm^{-}

[1]) in Figure S4(b) are assigned to –OH, benzene and $–C_2H_5$ vibrations, respectively. The result shows that the liquid of distillage is aromatic solvent oil.

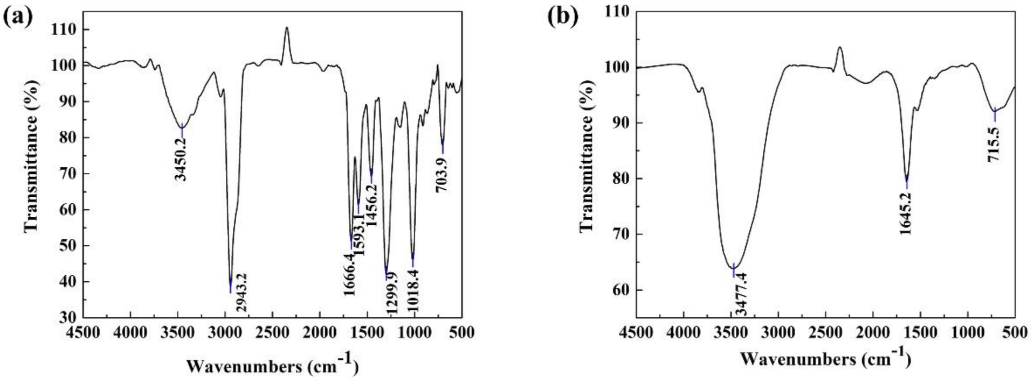

Figure S4. The infrared spectra of recovered products by distillation, (a) solid product and (b) liquid product.

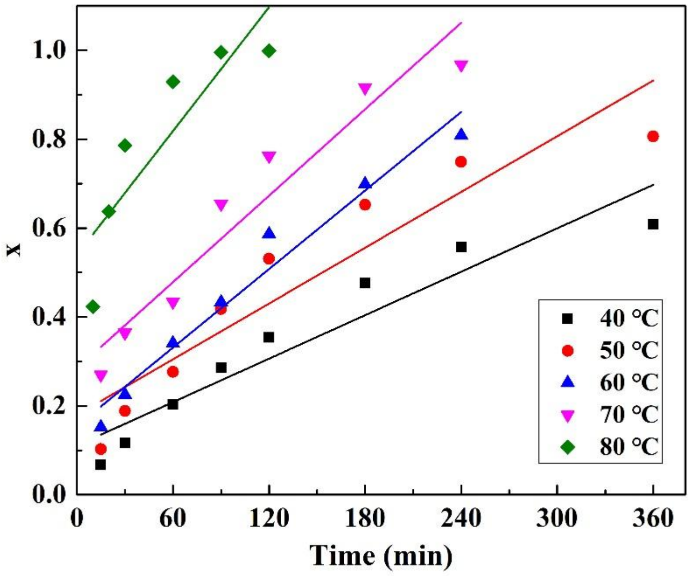

Figure S5. Plots of x vs t at different reaction temperatures.

Table S1. Kinetics parameters during the leaching process calculated by the mass transfer control model.

T	40 ℃	50 ℃	60 ℃	70 ℃	80 ℃
k	0.00163	0.00209	0.00295	0.00324	0.00464
R^2	0.8943	0.8601	0.9605	0.9162	0.7085

The lines at 40, 50, 80 ℃ in Figure S5 are non-linear as the correlation coefficients (R^2=0.8943, 0.8601 and 0.7085, respectively) are below 0.9, indicating the linearity is poor. This result shows that Pd leaching is not controlled by mass transfer.

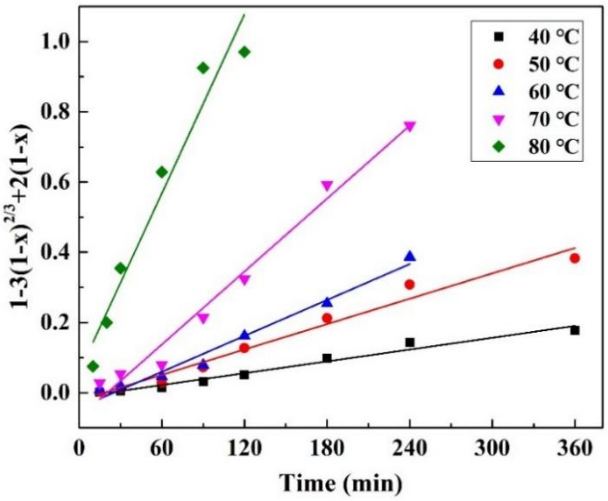

Figure S6. Plots of $1 - 3(1 - x)^{2/3} + 2(1 - x)$ vs t at different reaction temperatures.

Table S2. Kinetics parameters during the leaching process calculated by the ash layer diffusion model.

**T**	**40 ℃**	**50 ℃**	**60 ℃**	**70 ℃**	**80 ℃**
k	0.0028	0.0021	0.0030	0.0032	0.0059
R^2	0.9603	0.8601	0.9605	0.9162	0.9005

The lines at 50, 70, 80 ℃ in Figure S6 is non-linear as the correlation coefficients (R^2=0.8601, 0.9162 and 0.9005, respectively) are about 0.9, indicating the linearity is poor. This result shows that Pd leaching is not controlled by ash layer diffusion.

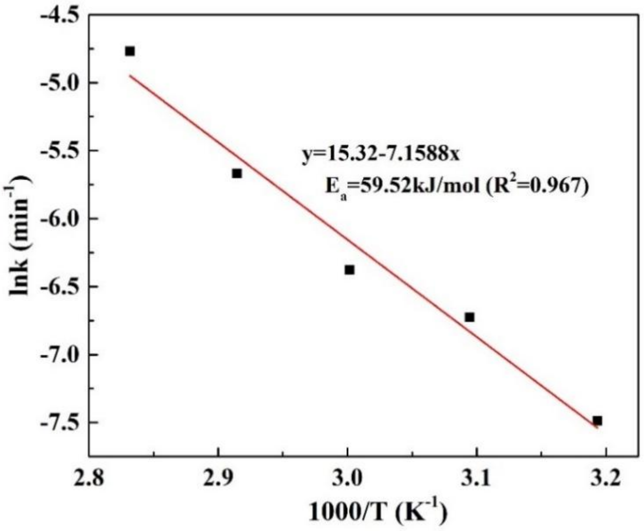

Figure S7. Arrhenius plot for the leaching of Pd from spent catalysts calculated by the ash layer diffusion model.

Moreover, the apparent activation energy calculated by the ash layer diffusion model was 59.52 kJ/mol, which was not within the reasonable range (10-20 kJ/mol) (Zhang et al. 2004).

Selective Adsorption of Pd by R_{410} Ion Exchange Resins.

R_{410} anion exchange resin was used for Pd absorption. Column experiments were conducted in a 5.0 cm diameter × 80 cm height Plexiglas tube in which R_{410} anion exchange resin was wet-packed. The leachate containing $PdCl_4^{2-}$ was poured into the tube. The flow rate of column effluent was controlled at 5.0 ml/min. After the leachate had passed through the column, it was flushed with deionized water until the column effluent was colorless. And then the resin was eluted with 40 g/L NH_4Cl + 8% NH_3 solution at a flow rate of 5 mL/min to desorb $[PdCl_4]^{2-}$. The resin was regenerated by being flushed with 20% NaOH solution, deionized waste, and 6.0 mol/L HCl solution in turn.

The Pd-containing solutions were from leaching processes under the condition of (1) Fe^{3+} 0-1.0 mol/L, HCl 2.0 mol/L, NaCl 4.0 mol/L, S/L of 1:5 at 80 °C for 2.0 h; (2) HCl 2.0-6.0 mol/L, NaCl 4.0 mol/L, S/L of 1:5 at 80 °C for 2.0 h. Table S3 shows the effect of Fe^{3+} on the absorption efficiency of Pd. It can be seen that R_{410} ion exchange resins exhibited a high absorption affinity to Pd. Pd absorption efficiency was over 97.5% when the concentration of Fe^{3+} was below 0.67 mol/L. As the Fe^{3+} concentration increased to 1.0 mol/L, the absorption efficiency decreased slightly to 93.37%. Table S4 shows the effect of H^+ on the absorption efficiency of Pd. Pd absorption efficiency varied from 97.98% to 99.41% as the concentrations of H^+ were between 2.0-6.0 mol/L, indicating that it has little influence on the sorption of Pd by R_{410} ion exchange resins.

Table S3. The effect of Fe^{3+} on the adsorption efficiency of Pd by R_{410} ion exchange resins.

Fe^{3+} (mol/L)	c_1 (mg/L)	v_1 (mL)	c_2 (mg/L)	v_2 (mL)	A (%)
0	255.6	200	0.829	426	99.31%
0.33	238.6	200	1.539	698	97.75%
0.67	269.5	200	2.891	440	97.64%
1.0	176. 0	200	3.340	699	93.37%

Table S4. The effect of H^+ on the adsorption efficiency of Pd by R_{410} ion exchange resins.

c (HCl) mol/L	c_1(g/L)	v_1 (mL)	c_2 (g/L)	v_2 (mL)	A(%)
2	247.6	200	1.300	770	97.98%
3	230.6	200	0.926	400	99.20%
4	258.3	200	1.885	521	98.10%
5	301.4	200	1.004	445	99.26%
6	288.0	200	0.723	468	99.41%

Adsorption efficiency of Pd is given below.

$$A = \frac{c_1 \times v_2 - c_2 \times v_2}{c_1 \times v_1} \times 100\%$$

where c_1 and c_2 are the concentrations of Pd in the leachate and tail liquid; v_1 and v_2 are the volumes of leachate and tail liquid. A is the adsorption efficiency of Pd.

Elution of Palladium from Loaded Ion Exchange Resins.

The Pd-loaded resins were stripped by using four different elution reagents: (i) 8.0% NH_3, (ii) 40 g/L NH_4Cl +8.0% NH_3, (iii) 1.0 mol/L thiourea and (iv) 2.0 mol/L NaOH. The Pd in the resins were selectively eluted at the rate of 5.0 mL/min. Following the 2.0h elution, the resins were washed with deionized water. The result was shown in Table A5. The eluents 1.0 mol/L thiourea and 2.0 mol/L NaOH were relatively ineffective in eluting Pd from the loaded R_{410} ion exchange resins. The elution

efficiency was less than 5% with the eluent solutions. The 8.0% NH_3 and 40 g/L NH_4Cl + 8.0% NH_3 reagents desorbed 64.27% and 94.73% of Pd, respectively. The result showed that 40 g/L NH_4Cl + 8.0% NH_3 has successfully stripped Pd from the loaded resins.

The elution of PGMs from selective ion exchange resins is rather difficult because of strong chemical bonds of adsorbed metal ions with functional groups on the resins. Thus, it is necessary to choose appropriate eluent reagents that can form more stable complexes with the PGM ions than the existing complexes in the resins. The mixture solution of 40 g/L NH_4Cl and 8.0% NH_3 eluent was found to be effective.

Table S5. Elution efficiency of Pd from loaded resins by using different reagents.

Elution agents	NH_3	NH_4Cl + NH_3	thiourea	NaOH
Elution rate (%)	64.27%	94.73%	3.48%	4.36%

Table S6. The desorption efficiency of Pd by NH_4Cl + NH_3.

c_0 (mg/L)	v_0 (mL)	c_1 (mg/L)	v_1 (mL)	c_2 (mg/L)	c_2 (mL)	Q
269.5	200	2.891	440	117.32	422	94.73%

Q is the elution efficiency of Pd by NH_4Cl + NH_3.

$$Q = \frac{c_2 \times v_2}{c_0 \times c_0 - c_1 \times v_1} \times 100\%$$

where c_0, c_1 and c_2 are the concentrations of leaching solution, after adsorption and after elution, respectively; v_0, v_1 and v_2 are the volumes of leaching solution, after adsorption and after elution, respectively.

Leaching Efficiency of Pd by Reusing Leaching Agent

After the leachate had passed through the column of anion exchange resins, The tail liquid containing large amounts of Fe^{3+}, H^+, Cl^-, and Fe^{2+} was reused as leaching agent. It is assumed that the amounts of Fe ions and Cl^- were equal to the leaching agent before leaching since their consumptions were less than 1%. 1.0 ml H_2O_2 was added to oxide Fe^{2+} into Fe^{3+}. The composition of leaching agent was adjusted by $HCl_{(aq)}$ (37%), $FeCl_3{\cdot}6H_2O$ and NaCl. Table S7 shows the leaching efficiency of Pd after 5 cycle times of reusing leaching agent.

Table S7. The leaching efficiency of Pd by reusing leaching agent.

Cycle number	1	2	3	4	5
c($PdCl_4^{2-}$) (mg/L)	374.8	397.0	385.5	375.29	389.9
v_1 (ml)	316	296	327	309	294
Leaching efficiency	99.53%	98.76%	98.53%	97.46%	96.31%

v_1 is the volume of the solution after filtration, which is large than 250 ml since deionized water has been added during filtration.

References

Zhang, J.Y., 2004. Physical chemistry of metallurgy (Chinese). Press of Metallurgy Industry, Beijing.
